# Self-Pulsations in Terahertz Quantum Cascade Lasers under Strong Optical Feedback: The Effect of Multiple Reflections in the External Cavity

**DOI:** 10.3390/s22218501

**Published:** 2022-11-04

**Authors:** Xiaoqiong Qi, Hui Yi Loh, Thomas Taimre, Karl Bertling, Dragan Indjin, Aleksandar D. Rakić

**Affiliations:** 1 School of Information Technology and Electrical Engineering, The University of Queensland, Brisbane, QLD 4072, Australia; 2 School of Mathematics and Physics, The University of Queensland, Brisbane, QLD 4072, Australia; 3 School of Electronic and Electrical Engineering, University of Leeds, Leeds LS2 9JT, UK

**Keywords:** terahertz quantum cascade lasers, strong optical feedback, multiple reflections, self-pulsations, rate equation model

## Abstract

We have recently reported the self-pulsation phenomenon under strong optical feedback in terahertz (THz) quantum cascade lasers (QCLs). One important issue, however, we left open: the effect of multiple round trips in the external cavity on the laser response to feedback. Our current analysis also casts additional light on the phenomenon of self-pulsations. Using only one external cavity round trip (ECRT) in the model has been the common approach following the seminal paper by Lang–Kobayashi in 1980. However, the conditions under which the Lang–Kobayashi model, in its original single-ECRT formulation, is applicable has been rarely explored. In this work, we investigate the self-pulsation phenomenon under multiple ECRTs. We found that the self-pulsation waveform changes when considering more than one ECRT. This we attribute to the combined effect of the extended external cavity length and the frequency modulation of the pulsation frequency by the optical feedback. Our findings add to the understanding of the optical feedback dynamics under multiple ECRTs and provide a pathway for selecting the appropriate numerical model to study the optical feedback dynamics in THz QCLs and semiconductor lasers in general.

## 1. Introduction

**Figure 1:**
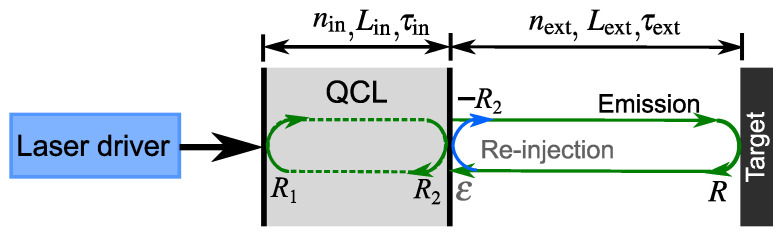
Model of a QCL under optical feedback with multiple external cavity round trips, where $R_{1}$, $R_{2}$, and *R* are the reflection coefficient of the laser facets and the external target, respectively, and ε is the reinjection coupling factor. The blue arrow indicates the reflection of the THz beam when it is trying to return to the laser cavity after being reflected from the external target, with the reflection coefficient $-R_{2}$.

Terahertz (THz) quantum cascade lasers (QCLs) have undergone rapid development since their demonstration in 2002 [1], showing high emission power [2], quantum-limited linewidth [3], ultra-broadband gain [4,5], and being able to operate at high temperatures of up to 250 K in pulse mode [6]. Due to the absence of the relaxation oscillations and the small linewidth enhancement factor in the THz QCLs, it was widely accepted that THz QCLs are ultra-stable against optical feedback [7,8]. Recently, we reported the first observations of self-pulsations in a single-mode THz QCL [9] and demonstrated THz imaging with a fixed-current point THz QCL by using the self-pulsation phenomenon [10] and the modulation effects of the self-pulsation dynamics on the self-mixing waveforms [11]. It was observed that the self-pulsation phenomenon caused by the beating of the external cavity modes occurs under strong feedback regimes. In this condition, there is more than one round-trip reflection and propagation of the laser beam in the external cavity due to the non-perfect reflection coefficient of the laser facet. This is illustrated in Figure 1 for a QCL under optical feedback; the blue arrow indicates the reflection of the THz beam at the outside of the laser facet when it is trying to return to the laser cavity after being reflected from the target (with the reflection coefficient $-R_{2}$). That is the start of the subsequent multiple round trips in the external cavity.

However, over the past four decades, using only one external cavity round trip (ECRT) in the model has been the commonly used approach following the seminal paper by Lang–Kobayashi in 1980 [7,8,12,13,14,15,16,17]. The boundary and applicability of the Lang–Kobayashi model in its original single-ECRT formulation has been rarely explored since its inception. Providing the justification for this assumption, and determining the conditions under which this approach is valid, is an important contribution to modeling the optical feedback in laser systems. The Lang–Kobayashi model only considers a single reflection in the external cavity and thus is only suitable to describe weak feedback regimes. Although most of the sensing and imaging applications using self-mixing effects in different types of semiconductor lasers typically operate in a weak feedback regime (the *C* parameter is less than 1) [18,19,20,21,22,23,24,25,26,27,28,28], the THz sensing and imaging applications based on the self-pulsation phenomenon requires QCLs operating under moderate and strong feedback regimes with the benefit of a fixed-current point (no modulation of the laser current or the external cavity parameters is required) [10]. However, the influence of multiple ECRTs, which emerges in the presence of moderate and strong optical feedback, on the self-pulsations and the laser response to the optical feedback in general are still unexplored so far using a more rigorous model. The strong feedback condition with multiple ECRTs is automatically satisfied for external cavity (EC) QCLs with emission frequency tunabilities because the front laser facet is anti-reflection (AR)-coated with the reflection coefficient lower than 2% [29,30,31] to make the laser more sensitive to optical feedback and to suppress the laser cavity modes.

In this work, we investigate the effects of multiple ECRTs on the self-pulsation properties through both the reduced rate equations (RREs) and the excess-phase equation model. It was found that both the oscillation amplitude and frequency of the self-pulsations increases with the optical feedback strength [9,10]. The maximum oscillation frequency of the self-pulsations from the first ECRT is the resonant frequency of the external cavity $f_{0}=c/\left(2n_{\mathrm{ext}}L_{\mathrm{ext}}\right)$ (defined as the fundamental frequency of self-pulsations), where $n_{\mathrm{ext}}$ and $L_{\mathrm{ext}}$ are the refractive index and the physical length of the external cavity and *c* is the light speed in vacuum. Now, when we consider more than one ECRT, for example, the *s*-th ECRT, the maximum self-pulsation oscillation frequency from the *s*-th ECRT reduces to $f_{0}/s$ due to the fact that the total delay for the laser beam after *s*-th ECRT is $s\tau_{\mathrm{ext}}$. However, due to the frequency modulation property, the self-pulsation frequency from the *s*-th ECRT is not exactly at the harmonic of the fundamental self-pulsation frequency due to the weaker optical feedback level from the following ECRT than that of the first. Furthermore, it was found that the self-pulsation waveform varies when considering more than one ECRT due to a combination effect of the extended external cavity length and the frequency modulation of the pulsation frequency by the optical feedback level. The required number of ECRTs in the model increases with the optical feedback strength. By introducing the normalized energy deviation (NED), we quantified the accuracy of the numerical model and found that for a general laser without AR coating, we would need to consider two ECRTs when the optical reinjection coupling coefficient ε is larger than −4 dB and three ECRTs when ε is larger than −2 dB to be below the threshold NED of 0.13%. However, for EC lasers with a typical $R_{2}$ of 2%, we need to consider two and three ECRTs when ε is larger than −19 dB and −17 dB, respectively. This means the tolerance of the reinjection coupling factor of the laser beam from the external cavity back into the laser cavity to consider more than one ECRT is 15 dB lower in EC lasers compared to that for a general laser without AR coatings. Our findings add to the understanding of the optical feedback dynamics in THz QCLs under multiple ECRTs and provide the justification for selecting the appropriate numerical model to study the optical feedback dynamics in THz QCLs and semiconductor lasers in general.

## 2. Theoretical Model

The set of single-mode RREs with optical feedback terms for a THz QCL by involving up to the *N*-th order ECRT are shown below with Equations (1)–(4), where the feedback coupling coefficient of the *s*-th ECRT $\kappa_{s}$ is defined as: $\kappa_{s}=\varepsilon^{s}\sqrt{R/R_{2}}\left(1-R_{2}\right){\left(-\sqrt{RR_{2}}\right)}^{s-1}$. The terms S(t) and φ(t) are the photon population and the phase of the electric field, respectively, while $N_{3}\left(t\right)$ and $N_{2}\left(t\right)$ represent the carrier populations in the upper and lower laser levels (ULL/LLL) of the active cavity. Once the equations are solved, the time traces of the emission output power can be calculated by $P_{\mathrm{out}}\left(t\right)\;=\;\eta_{0}\hbar \omega S\left(t\right)/\tau_{p}$, where $\eta_{0}=a_{m}/\left(2a_{\mathrm{total}}\right)$ is power output coupling coefficient, where $a_{m}=\ln{\left(R_{2}\right)}^{-1}/L_{\mathrm{in}}$ is the mirror loss of the laser cavity and $a_{\mathrm{total}}$ is the total loss in the laser cavity, including the mirror loss and waveguide loss. The meaning and value of the other parameters are summarized in Table 1 if not described elsewhere.


(1) $$\begin{array}{c} \frac{dS(t)}{dt}=MG\left(N_{3}\left(t\right)-N_{2}\left(t\right)\right)S\left(t\right)+\frac{M\beta_{\mathrm{sp}}N_{3}\left(t\right)}{\tau_{\mathrm{sp}}}-\frac{S(t)}{\tau_{p}}\\ +\underset{\mathrm{The}\;\mathrm{sum}\;\mathrm{of}\;\mathrm{multiple}\;\mathrm{feedback}\;\mathrm{terms}\;(\mathrm{from}\;1\;\mathrm{to}\;N)}{\underbrace{\sum_{s=1}^{N}\frac{2\kappa_{s}}{\tau_{\mathrm{in}}}\sqrt{S\left(t\right)S\left(t-s\tau_{\mathrm{ext}}\right)}\cos\left(s\omega_{\mathrm{th}}\tau_{\mathrm{ext}}+\varphi (t)-\varphi (t-s\tau_{\mathrm{ext}})\right)}}, \end{array}$$

(2) dφ(t)dt=α2MG(N3(t)−N2(t))−1τp=−∑s=1NκsτinS(t−sτext)S(t)sinsωthτext+φ(t)−φ(t−sτext)⏟Thesumofmultiplefeedbackterms(from1toN),

(3) $$\frac{dN_{3}\left(t\right)}{dt}=\frac{\eta_{3}}{q}\;I\left(t\right)-G\left(N_{3}\left(t\right)-N_{2}\left(t\right)\right)\;S\left(t\right)-\frac{N_{3}\left(t\right)}{\tau_{3}}\;,$$

(4) $$\frac{dN_{2}\left(t\right)}{dt}=\frac{\eta_{2}}{q}\;I\left(t\right)+G\left(N_{3}\left(t\right)-N_{2}\left(t\right)\right)\;S\left(t\right)+\frac{N_{3}\left(t\right)}{\tau_{32}}+\frac{N_{3}\left(t\right)}{\tau_{\mathrm{sp}}}-\frac{N_{2}\left(t\right)}{\tau_{21}}\;,$$

The steady state of the phase rate equation, namely the excess-phase equation, by involving multiple ECRTs from 1 to *N* is as follows:

(5) $$\begin{array}{c} \varphi_{FB}-\varphi_{s}+\sum_{s=1}^{N}C_{s}\sin\left({s\varphi }_{\mathrm{FB}}+\arctan\alpha \right)=0\;. \end{array}$$

## 3. Results

### 3.1. Effects of Each External Cavity Round Trip on Self-Pulsations Separately

**Figure 2:**
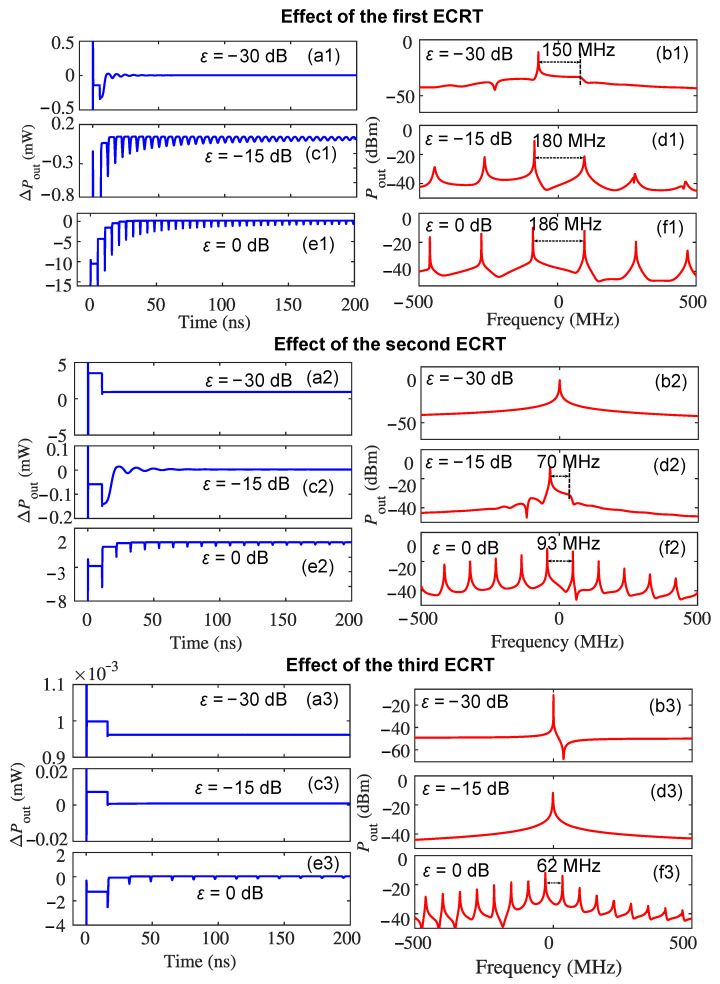
The effects of the first, second, and third ECRT on the self-pulsation dynamics in a THz QCL under varying optical feedback levels, respectively: (**a**,**c**,**e**) are the AC component of the emission power $\Delta P_{\mathrm{out}}$ when ε is −30 dB, −15 dB, and 0 dB, respectively; (**b**,**d**,**f**) are the emission spectrum (offset to the emission frequency 2.752 THz) when ε is −30 dB, −15 dB, and 0 dB, respectively.

The optical feedback dynamics in THz QCLs are very different from that in MIR-QCLs due to their smaller value of the linewidth enhancement factor. The nonlinear dynamics that include the typical five feedback regimes found in diode lasers [13] have been observed in MIR-QCLs [17,32]. However, only frequency splitting and associated self-pulsations were observed in optically reinjected THz QCLs [9], where we only considered the first ECRT in the theoretical models. Here, we investigate the effects of the second and the third ECRT after the first one on the self-pulsation dynamics. It is estimated that the maximum oscillation frequency of the self-pulsations induced by the *s*-th ECRT is 1/s times the fundamental frequency of the self-pulsations $f_{0}$ (as defined in the Introduction) due to the fact that the external cavity length is extended by a factor of *s* for the *s*-th ECRT. However, because the oscillation frequency is also determined by the optical feedback strength, it increases with the optical feedback strength and only equals to the resonant frequency of the external cavity with 100% optical feedback (ε = 0 dB) [9]. Because the optical feedback strengths of the later ECRTs are always weaker than the first one, this results in the beating frequencies in the laser cavity from the *s*-th ECRT being not exactly at the harmonics of the fundamental frequency $f_{0}$. As demonstrated in Figure 2, the oscillation frequency of the self-pulsation dynamics from the first ECRT increases with the reinjection coupling factor ε and is 150 MHz, 180 MHz, and 186 MHz when ε is −30 dB, −15 dB, and 0 dB, respectively. However, at the same ε, the self-pulsation dynamics have a decreasing amplitude from the first to the second and to the third ECRT due to the decreasing optical feedback strengths. At ε=−30 dB, the single-mode emission frequency from the laser splits to the external cavity modes in Figure 2(b1) (10 dB linewidth of the central mode: 4 MHz), but only single-mode linewidth broadening is observed from the second ECRT in Figure 2(b2) (10 dB linewidth: 8 MHz) and a narrower linewidth broadening from the third ECRT in Figure 2(b3) (10 dB linewidth: 3 MHz). The single frequency in Figure 2(b2,b3) indicates the emission frequency of the single-mode QCL with the feedback level of −30 dB, namely the CW emission at steady state for the slowly varying envelope of the electric field. At ε=−15 dB, the oscillation frequency of the self-pulsations for the first ECRT is 180 MHz in Figure 2(d1). However, the self-pulsations from the second ECRT is at 70 MHz (Figure 2(d2)), which is smaller than 180 MHz/2 due to the weaker optical feedback strength for the second ECRT. Nevertheless, under the strongest optical feedback condition when ε is 0 dB (ε=1), the difference between $\kappa_{1}$ and $\kappa_{2}$ is much smaller than the case when ε is −15 dB, so the oscillation frequencies of the self-pulsations from the first ECRT (186 MHz) reduces to its half-frequency of 93 MHz [Figure 2(f2)] and 1/3 of the fundamental frequency (62 MHz) (Figure 2(f3)) for the second and third ECRT, respectively. It should be noted that the self-pulsations are transient instabilities in the THz QCLs with small values of the linewidth enhancement factor. The stronger the optical feedback, the longer the transient instabilities last [9].

### 3.2. Effects of Multiple External Cavity Round Trips on Self-Pulsations Simultaneously

**Figure 3:**
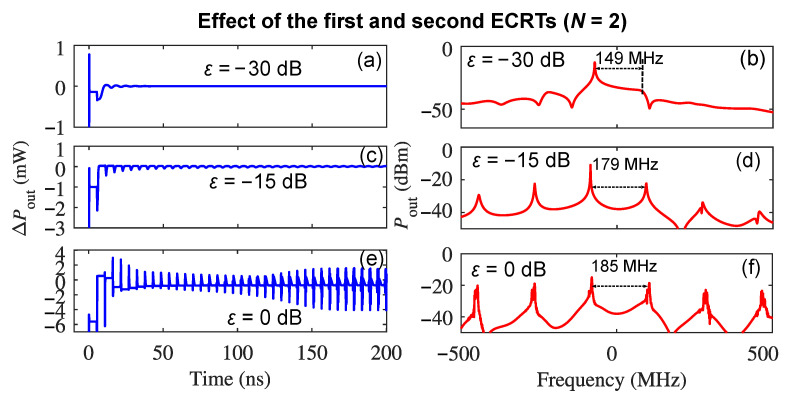
The cumulative effects of the first and second ECRTs on the self-pulsation dynamics in a THz QCL under optical feedback: (**a**,**c**,**e**) are the AC component of the emission power $\Delta P_{\mathrm{out}}$ when ε is −30 dB, −15 dB, and 0 dB, respectively; (**b**,**d**,**f**) are the emission spectrum (offset to the emission frequency 2.752 THz) when ε is −30 dB, −15 dB, and 0 dB, respectively.

Once we have understood the effect of each individual ECRT on the self-pulsation dynamics separately, we now demonstrate the overall effects of *N* ECRTs simultaneously on the self-pulsations and determine how many ECRTs are needed to be involved in the theoretical model to describe the self-pulsation properties under varying optical feedback strengths. The self-pulsation dynamics and the corresponding spectrum by considering the first and second ECRTs simultaneously (N=2) are shown in Figure 3, where (a), (c), and (e) are the AC component of the emission power $\Delta P_{\mathrm{out}}$, and (b), (d), and (f) are the emission spectrum when ε is −30 dB, −15 dB, and 0 dB, respectively. Compared with the results in Figure 2 with the first ECRT only, it was found that the self-pulsation waveform when *N* equals to 2 is very similar when ε is −30 dB and −15 dB, respectively. However, when ε is 0 dB, the time-domain waveform when N=2 is distinct from that with the first ECRT only, which comes from the additional phase shift of π from the 2nd ECRT due to $C_{2}$ < 0. Nevertheless, the emission spectrum of the complex field envelope when N=2 is still dominated by the effect of the first ECRT as shown in Figure 3, where the oscillation frequency of the self-pulsations with ε at −30 dB, −15 dB, and 0 dB is 149 MHz, 179 MHz, and 185 MHz, respectively, which is only a 1 MHz difference compared with the corresponding result shown in Figure 2(d1,f1).

**Figure 4:**
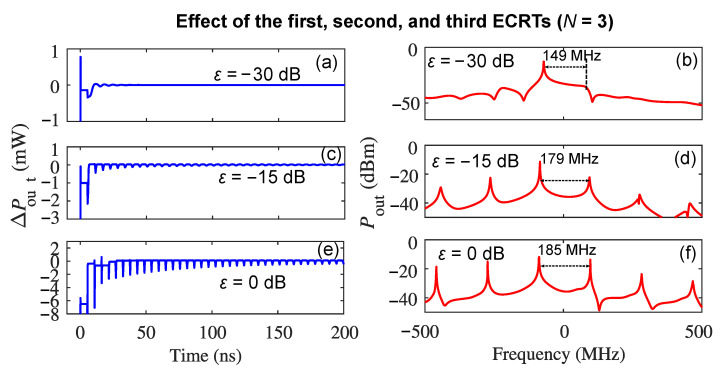
The cumulative effects of the first, second, and third ECRTs on the self-pulsation dynamics in a THz QCL under optical feedback: (**a**,**c**,**e**) are the AC component of the emission power $\Delta P_{\mathrm{out}}$ when ε is −30 dB, −15 dB, and 0 dB, respectively; (**b**,**d**,**f**) are the emission spectrum (offset to the emission frequency 2.752 THz) when ε is −30 dB, −15 dB, and 0 dB, respectively.

In addition, we simulated the self-pulsations using the RREs with N=3 in Equations (1) and (2); the results are shown in Figure 4, where (a), (c), and (e) are the time-domain waveform and (b), (d), and (f) are the frequency-domain spectra of the self-pulsations when ε is −30, −15, and 0 dB, respectively. It was observed that while the spectra has the same mode spacing at each ε when compared to the case when N=2, the self-pulsation waveform when ε = 0 is simplified in Figure 2(e1) due to involving the 3rd ECRT with a positive $C_{3}$, which helps the positive $C_{1}$ from the first ECRT to compensate for the effect of the negative $C_{2}$ and dominate the self-pulsation dynamics. It was noted that the optical feedback dynamics are dependent on the value of the linewidth enhancement factor (α) of the laser [9]. For this particular design of the THz QCL used in our system, we extracted α experimentally and it was −0.1 [23], which agrees well with that reported by [5,33,34]. Although α for THz QCLs varies from −0.2 to 0.5, depending on the driving current and the aperture within the external beam path [33], the effects of the multiple ECRTs on the self-pulsation dynamics demonstrated here would not change qualitatively as long as the value of α is within the reported range for the THz QCLs.

### 3.3. Normalized Energy Deviation with a Varying Feedback Level

In order to quantitatively evaluate the difference between the self-pulsation waveforms under two conditions, we define the NED as follows:

(6) $$\begin{array}{c} \mathrm{NED}=\frac{\int |P_{\mathrm{out}}\left(t\right)-P_{\mathrm{out}0}{\left(t\right)|}^{2}dt}{\int |P_{\mathrm{out}0}{\left(t\right)|}^{2}dt}\;. \end{array}$$

where $P_{\mathrm{out}}\left(t\right)$ and $P_{\mathrm{out}0}\left(t\right)$ are the laser output power with the optical feedback condition under study and the reference output power, respectively. The emission power can be simulated from the set of RREs.

**Figure 5:**
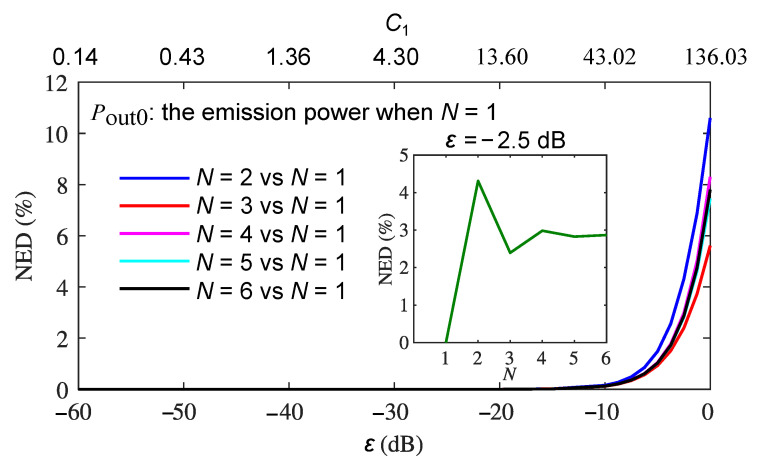
NED between the emission power with varying *N* and the emission power reference when N=1 as a function of reinjection coupling factor ε and the feedback parameter $C_{1}$.

For the purpose of showing the NED estimation changes with multiple ECRTs relative to the first ECRT when N=1, we use the emission output power with the first ECRT only (N=1) as the reference $P_{\mathrm{out}0}\left(t\right)$, and the NED of the emission output power when N=2, 3, 4, 5, and 6 from $P_{\mathrm{out}0}\left(t\right)$ is calculated as a function of ε and $C_{1}$ as shown in Figure 5. As expected, the NED for all *N* increases with the optical feedback level ε. At a fixed ε, such as −2.5 dB, it was observed that the value of NED repeats the alternating of a decreasing and increasing process when the round trip increases from 2 to 6, as plotted in the inset of Figure 5. The reason behind this phenomenon is that the feedback coupling coefficient $\kappa_{s}$ and $C_{s}$ parameter are negative when *N* is an even number (2, 4, 6, etc.). This leads to an additional phase shift of π of the returned beam, and the self-mixing between that particular returned beam and the existing beam results in more complicated self-pulsation waveforms as shown in Figure 3e, which increases the value of the corresponding NED.

**Figure 6:**
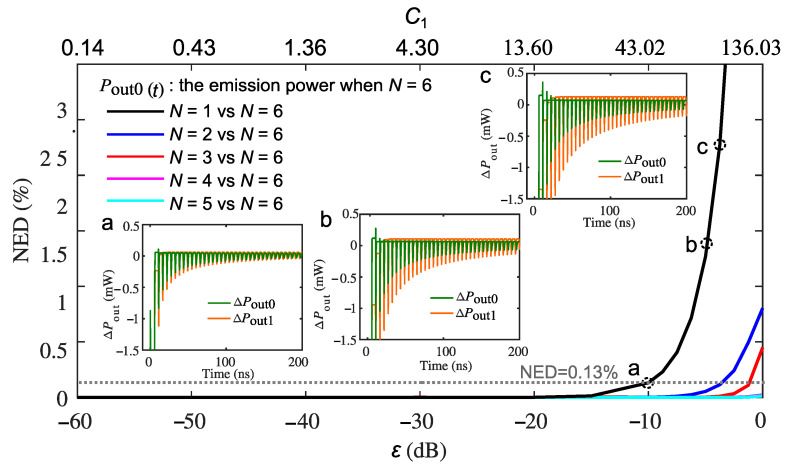
NED between the emission power with varying *N* and the emission power reference when N=6 as a function of reinjection coupling factor ε and the feedback parameter $C_{1}$. The AC component of the self-pulsation waveform of the reference emission power ($\Delta P_{\mathrm{out}0}$) and that with *N* equals to 1 ($\Delta P_{\mathrm{out}1}$) at Point a (ε=−10 dB, $C_{1}=43.02$), b (ε=−5 dB, $C_{1}=76.50$), and c (ε=−3.75 dB, $C_{1}=88.34$) along the black curve (when N=1) are chosen and shown with green and orange curves in inset a, b, and c, respectively. The dashed grey line indicates the threshold of NED = 1.3% to involve more than one ECRT in the RRE model.

On the other hand, if using the emission output power when N=6 as the reference $P_{\mathrm{out}0}\left(t\right)$ (which are the most accurate results under multiple ECRTs), the NED of the emission output power when N=1,2,3,4, and 5 from $P_{\mathrm{out}0}\left(t\right)$ is calculated and shown in Figure 6 in the black, blue, red, cyan, and magenta curves, respectively. The higher the value of *N*, the smaller the value of NED as expected. In addition, in order to relate the value of NED with the self-pulsation waveform differences, three pairs of the time-domain self-pulsation waveform at Point a (ε=−10 dB, $C_{1}=43.02$), b (ε=−5 dB, $C_{1}=76.50$)), and c (ε=−3.75 dB, $C_{1}=88.34$) along the black curve (when N=1) are chosen and shown in inset a, b, and c in Figure 6, respectively. The green and orange curves in each pair of the inset are the AC component of the reference emission power ($\Delta P_{\mathrm{out}0}$) and that with *N* equals to 1 ($\Delta P_{\mathrm{out}1}$), respectively. The NED values at Point a, b, and c are 0.13 %, 1.26 %, and 2.28 %, respectively. When the strength of the optical feedback grows, more ECRTs have to be involved, and the precise number of ECRTs is dependent on the acceptable NED. Because we can see the time-domain waveform between *N* = 1 and *N* = 6 starts deviating from Point a with NED = 0.13%, we used NED = 0.13% here as a threshold to show how many ECRTs have to be involved with a varying feedback level. As indicated by the dashed grey line in Figure 6, we would need to consider two and three ECRTs when the optical reinjection coupling coefficient ε is larger than −4 dB and −2 dB, respectively.

It is worth noting that the results shown in Figure 5 and Figure 6 apply to general QCLs without any coatings on the laser facets where the reflection coefficient is determined only by the reflective index of the semiconductor material of the active region of the laser (3.3 in this case). However, for EC QCLs with an AR coating on the front facet of the laser chip, the reflection coefficient $R_{2}$ is reduced to lower than 2% [29,31] and the transmittance of the light beam coupled into the external cavity is significantly enhanced. In this case, the feedback coupling coefficient κ is greatly increased. This makes EC QCLs a platform operating under strong feedback regimes. In this case, we need to involve more than one ECRT with lower values of the reinjection coupling factor, which correspond to higher values of the total loss that the laser beam experiences in the external cavity. For example, when $R_{2}$ reduced to a typical value of 2%, κ, $C_{1}$ increases to 5.8 times larger. Therefore, the tolerance to consider more than one ECRT reduces by 15 dB. If we still use NED = 0.13% as the threshold, when $R_{2}$ is 2% (as is typical for EC lasers), we need to consider two and three ECRTs when the optical reinjection coupling coefficient ε is larger than −19 dB and −17 dB, respectively.

## 4. Excess-Phase Equation Analysis

**Figure 7:**
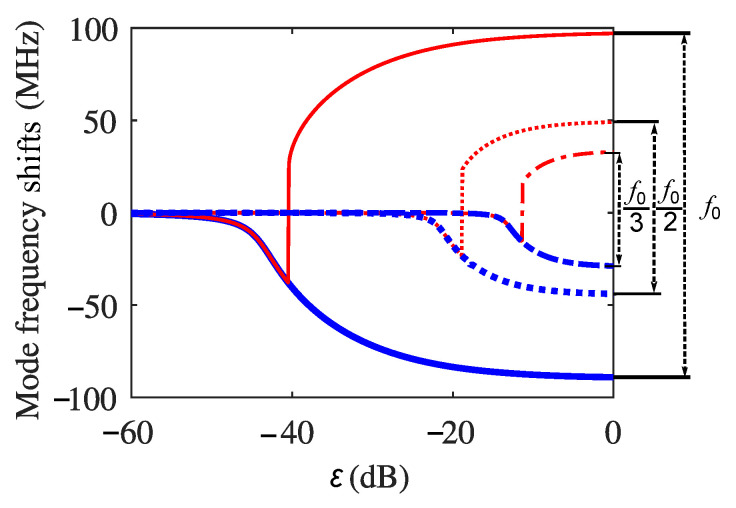
The fundamental (blue) and higher-order (red) solutions of the excess-phase equations by involving the first ECRT (the solid lines), the second ECRT (the dotted lines), and the third ECRT (the dashed dotted lines) separately in the THz QCL with optical feedback, where $L_{\mathrm{ext}}=0.8$ m.

In order to analyze the varying of the oscillation frequency of the self-pulsation dynamics with a continuous varying optical feedback level, we solved the excess-phase equation with multiple ECRTs, Equation (5), using the first, second, and third ECRT separately. The details of solving the excess phase equations under different conditions can be found in Appendix A. The results are shown in Figure 7, where the mode frequency shift of the fundamental mode and the adjacent first-order external cavity mode are shown as the blue and red curves, respectively. The solid lines, dotted lines, and the dash-dotted lines are the results for the first ECRT, the second ECRT, and the third ECRT, respectively. As shown in Figure 7, the oscillation frequency of the self-pulsations when N=1 and under the strongest optical feedback level when ε=0 dB is the fundamental frequency $f_{0}$, the mode spacing of the second ECRT under the same ε of 0 dB is $f_{0}/2$ and that of the third ECRT is $f_{0}/3$, which is consistent with the conclusions we draw from the simulation results obtained by solving the RREs (Figure 2(f1–f3)).

**Figure 8:**
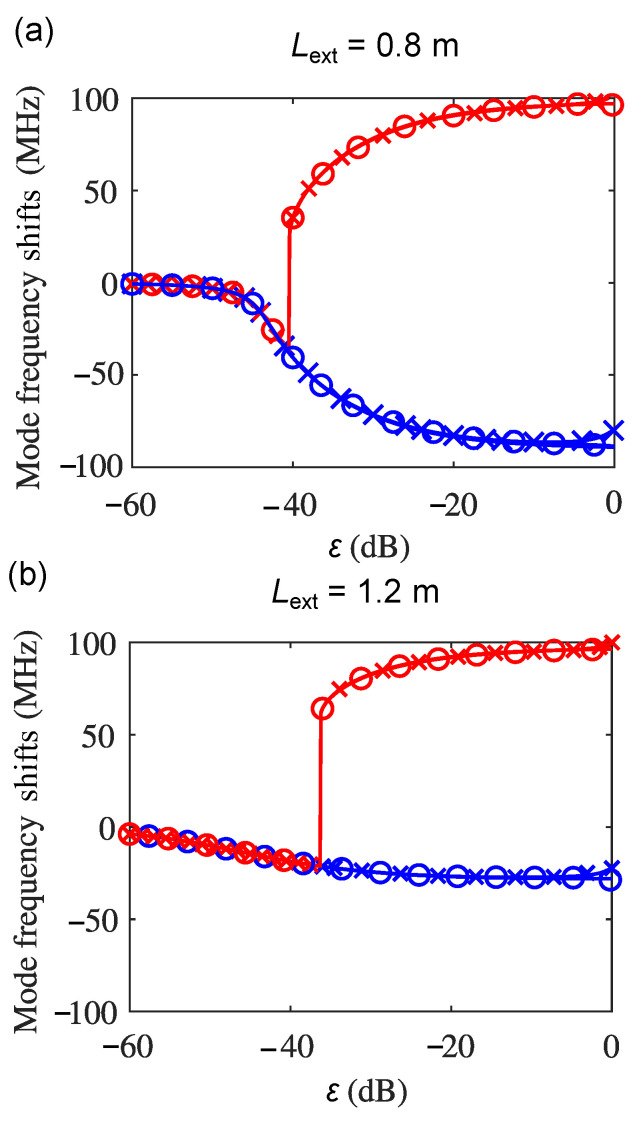
The mode split and shift in a THz QCL with optical feedback where (**a**) $L_{\mathrm{ext}}$ = 0.8 m and (**b**) $L_{\mathrm{ext}}$ = 1.2 m. In both figures, the fundamental (blue) and higher-order (red) solutions of the excess-phase equations when N=1 (the solid lines), N=2 (the solid line with cross markers), and N=3 (the solid line with circle markers).

Furthermore, we solved the excess-phase equations with multiple ECRTs simultaneously when N=1,2, and 3, and the mode frequency shifts of the fundamental mode and the first-order external cavity mode with ε under these three conditions are shown in solid lines, solid line with cross markers, and the solid line with the circle markers, respectively, in Figure 8, where (a) depicts the results with $L_{\mathrm{ext}}$ at 0.8 m and (b) shows the results for $L_{\mathrm{ext}}$ of 1.2 m. It can be found that the phase solutions of the fundamental mode and the first-order external cavity mode when N=2 and 3 are nearly overlapping with the results when N=1. This further proves that the self-pulsation dynamics in the frequency domain (due to frequency splitting) obtained from the first ECRT are dominant in the results with more than one ECRT. Because there is only one type of the optical feedback dynamics that was observed in the THz QCLs, namely the self-pulsation dynamics, the relevant conclusion does not change with the external cavity length, as shown in Figure 8b.

## 5. Conclusions

The self-pulsation phenomenon observed in THz QCLs under a strong feedback region recently triggered the study of an appropriate theoretical model for a THz QCL operating under this region. Furthermore, the effect of multiple round trips in the external cavity on self-pulsations, and on the laser response to optical feedback in general, is not explored. In this work, we investigate the effects of more than one ECRT on the self-pulsations. It is found that although theoretically the *s*-th ECRT creates the self-pulsations at a harmonic frequency (1/s of the fundamental frequency of the self-pulsations from the first ECRT $f_{0}$), in reality it is always smaller than $f_{0}/s$ due to the fact that the oscillation frequency of the self-pulsations is also dependent on the optical feedback strength and the optical feedback strength is always getting weaker after the first round trip (due to the attenuation and non-perfect reflections in the external cavity). Therefore, the consequence of the self-pulsation frequency with multiple ECRTs is the competition between the effects of the external cavity length and the optical feedback strength from each round trip. In addition, the time-domain waveform of the self-pulsations under the even number of ECRTs is more complicated than that from the odd number of ECRTs due to the negative *C* coefficients of the even number of ECRTs. Through extensive numerical simulations with RREs up to the sixth order of ECRT terms, we provide a chart with a defined normalized energy deviation of the laser emission power under up to a fifth-order ECRT as a function of the feedback strengths. It is found that the number of ECRTs needs to be included in the RREs depending on the feedback parameter *C*. For a general laser without an AR coating, we would need to consider two ECRTs when the optical reinjection coupling coefficient ε is larger than −4 dB and three ECRTs when ε is larger than −2 dB to be below the threshold NED of 0.13%. However, for EC lasers with a typical $R_{2}$ of 2%, we need to consider two and three ECRTs when ε is larger than −19 dB and −17 dB, respectively.

THz QCLs are a unique platform to study the purely external cavity mode-induced optical feedback dynamics due to the absence of the relaxation oscillations. However, the oscillations due to the beating of the external cavity resonant modes along with the amplitude and frequency modulation properties exist in both THz QCLs and other semiconductor lasers. Because the Lang–Kobayashi model we used in this work applies to general semiconductor lasers, the effects of multiple ECRTs on external cavity modes-induced optical feedback dynamics explored in this work is expected to apply to general semiconductor lasers.

## Figures and Tables

**Table 1:** Parameters used in Equations (1)–(5).

Parameter	Value
$\eta_{3}$—Injection efficiency into ULL	54.41%
$\eta_{2}$—Injection efficiency into LLL	1.65%
*I*—Drive current	1.2 A
$\tau_{3}$—Total carrier lifetime in ULL	$5.0\times 10^{-12}\;s$
$\tau_{32}$—Non-radiative relaxation time from ULL to LLL	$1.76\times 10^{-10}\;s$
$\tau_{2}$—Total carrier lifetime in LLL	$2.1\times 10^{-11}\;s$
$\tau_{\mathrm{sp}}$—Spontaneous emission lifetime	$1.0\times 10^{-6}\;s$
$\tau_{p}$—Photon lifetime	$9.02\times 10^{-12}\;s$
*G*—Gain factor	$2.3\times 10^{4}\;s^{-1}$
*M*—Number of periods in active cavity	90
$\beta_{\mathrm{sp}}$—Spontaneous emission factor	$1.627\times 10^{-4}$
$\omega_{\mathrm{th}}$—Emission frequency with no optical feedback	$1.73\times 10^{13}\;\mathrm{rad}/s$
$L_{\mathrm{ext}}$—External cavity length	0.8 m
$n_{\mathrm{ext}}$—Refractive index of external cavity	1.00
$\tau_{\mathrm{ext}}$—Round-trip time of the external cavity, $\tau_{\mathrm{ext}}=2L_{\mathrm{ext}}n_{\mathrm{ext}}/c$	$5.34\times 10^{-9}\;s$
$L_{\mathrm{in}}$—Laser cavity length	2 mm
$n_{\mathrm{in}}$—Refractive index of active region	3.3
$\tau_{\mathrm{in}}$—Round-trip time of laser cavity, $\tau_{\mathrm{in}}=2L_{\mathrm{in}}n_{\mathrm{in}}/c$	4.403 $\times 10^{-11}\;s$
$\kappa_{s}$—Feedback coupling coefficient of the s-th ECRT, $\kappa_{s}=\varepsilon^{s}\sqrt{R/R_{2}}\left(1-R_{2}\right){\left(-\sqrt{RR_{2}}\right)}^{s-1}$	Varies
ε—Reinjection coupling factor	Varies
*R*—Reflection coefficient of external target	0.7
$R_{1}$, $R_{2}$—Reflection coefficient of laser facets	0.2861
α—Linewidth enhancement factor	−0.1 [23]
$C_{s}$—Feedback parameter of the s-th ECRT, $C_{s}=\kappa_{s}\tau_{\mathrm{ext}}\sqrt{1+\alpha^{2}}/\tau_{\mathrm{in}}$	Varies
*q*—Elementary charge	1.602 $\times 10^{-19}\;C$
*c*—Speed of light in vacuum	299,792,458 m s${}^{-1}$

## Data Availability

The data presented in this study are available on request from the corresponding author.

## Appendix A

When solving the excess-phase equations, we need to find the varying range of the phases $\varphi_{\min}$ and $\varphi_{\max}$ and the possible values of *m* where a valid solution exists by considering the lower and upper bounds for it, namely $m_{\mathrm{lower}}$ and $m_{\mathrm{upper}}$, as defined in [20]. For the excess-phase equation involving the first ECRT only:

$$\begin{array}{c} \varphi_{\min}=\left(2m+1\right)\pi +\arccos\left(\frac{1}{C_{1}}\right)-\arctan\alpha ,\;\\ \varphi_{\max}=\left(2m+3\right)\pi -\arccos\left(\frac{1}{C_{1}}\right)-\arctan\alpha \\ m_{\mathrm{lower}}=\left\lceil \frac{\varphi_{s}+\arctan\alpha +\arccos\left(\frac{1}{C_{1}}\right)}{2\pi }-\frac{\sqrt{C_{1}^{2}-1}}{2\pi }-\frac{3}{2}\right\rceil \\ m_{\mathrm{upper}}=\left\lfloor \frac{\varphi_{s}+\arctan\alpha -\arccos\left(\frac{1}{C_{1}}\right)}{2\pi }-\frac{\sqrt{C_{1}^{2}-1}}{2\pi }-\frac{1}{2}\right\rfloor \end{array}$$

For the excess-phase equation involving the second ECRT only:

$$\begin{array}{c} \varphi_{\min}=\frac{2m\pi +\arccos\left(\frac{1}{2C_{2}}\right)-\arctan\alpha }{2},\;\\ \varphi_{\max}=\frac{\left(2m+1\right)\pi +\arccos\left(\frac{1}{2C_{2}}\right)-\arctan\alpha }{2}\\ m_{\mathrm{lower}}=\left\lceil \frac{{2\varphi }_{s}+\arctan\alpha -\arccos\left(\frac{1}{2C_{2}}\right)}{2\pi }-\frac{\sqrt{4C_{2}^{2}-1}}{2\pi }-\frac{1}{2}\right\rceil \\ m_{\mathrm{upper}}=\left\lfloor \frac{{2\varphi }_{s}+\arctan\alpha -\arccos\left(\frac{1}{2C_{2}}\right)}{2\pi }+\frac{\sqrt{4C_{2}^{2}-1}}{2\pi }\right\rfloor \end{array}$$

For the excess-phase equation involving the third ECRT only:

$$\begin{array}{c} \varphi_{\min}=\frac{\left(2m+1\right)\pi +\arccos\left(\frac{1}{3C_{3}}\right)-\arctan\alpha }{3},\;\\ \varphi_{\max}=\frac{\left(2m+3\right)\pi -\arccos\left(\frac{1}{3C_{3}}\right)-\arctan\alpha }{3}\\ m_{\mathrm{lower}}=\left\lceil \frac{{3\varphi }_{s}+\arctan\alpha +\arccos\left(\frac{1}{3C_{3}}\right)}{2\pi }-\frac{\sqrt{9C_{3}^{2}-1}}{2\pi }-\frac{3}{2}\right\rceil \\ m_{\mathrm{upper}}=\left\lfloor \frac{{3\varphi }_{s}+\arctan\alpha -\arccos\left(\frac{1}{3C_{3}}\right)}{2\pi }+\frac{\sqrt{9C_{3}^{2}-1}}{2\pi }-\frac{1}{2}\right\rfloor \end{array}$$

For the situation with more than one ECRT, we used the formula for $\varphi_{\min}$, $\varphi_{\max}$, $m_{\mathrm{lower}}$, and $m_{\mathrm{upper}}$, the same as for the first ECRT only.
